# Bovine Interferon Lambda Is a Potent Antiviral Against SARS-CoV-2 Infection *in vitro*

**DOI:** 10.3389/fvets.2020.603622

**Published:** 2020-11-06

**Authors:** Nancy Patricia Cardoso, Florencia Celeste Mansilla, Estefanía Benedetti, Cecilia Soledad Turco, Lucas José Barone, Javier Alonso Iserte, Ivana Soria, Elsa Baumeister, Alejandra Victoria Capozzo

**Affiliations:** ^1^Instituto de Virología e Innovaciones Tecnológicas IVIT, Consejo Nacional de Investigaciones Científicas y Técnicas (CONICET) – Instituto Nacional de Tecnología Agropecuaria (INTA), Hurlingham, Argentina; ^2^Servicio Virosis Respiratorias, Instituto Nacional de Enfermedades Infecciosas, INEI–ANLIS “Dr. Carlos G. Malbrán,” Buenos Aires, Argentina; ^3^Structural Bioinformatics Group, Fundación Instituto Leloir, Buenos Aires, Argentina

**Keywords:** COVID-19, bovine IFN-λ, antivirals, respiratory viruses, biotherapeutic agent

## Abstract

Interferon lambda (IFN-λ) is an antiviral naturally produced in response to viral infections, with activity on cells of epithelial origin and located in the mucosal surfaces. This localized activity results in reduced toxicity compared to type I IFNs, whose receptors are ubiquitously expressed. IFN-λ has been effective in the therapy of respiratory viral infections, playing a crucial role in potentiating adaptive immune responses that initiate at mucosal surfaces. Human IFN-λ has polymorphisms that may cause differences in the interaction with the specific receptor in the human population. Interestingly, bovine IFN-λ3 has an *in silico*-predicted higher affinity for the human receptor than its human counterparts, with high identity with different human IFN-λ variants, making it a suitable antiviral therapeutic candidate for human health. Here, we demonstrate that a recombinant bovine IFN-λ (rbIFN-λ) produced in HEK-293 cells is effective in preventing SARS-CoV-2 infection of VERO cells, with an inhibitory concentration 50% (IC50) between 30 and 50 times lower than that of human type I IFN tested here (α2b and β1a). We also demonstrated the absence of toxicity of rbIFN-λ in human PBMCs and the lack of proinflammatory activity on these cells. Altogether, our results show that rbIFN-λ is as an effective antiviral potentially suitable for COVID-19 therapy. Among other potential applications, rbIFN-λ could be useful to preclude virus dispersion to the lungs and/or to reduce transmission from infected people. Moreover, and due to the non-specific activity of this IFN, it can be potentially effective against other respiratory viruses that may be circulating together with SARS-CoV-2.

## Introduction

Interferons (IFNs) are antiviral cytokines produced by almost any cell type upon recognition of viral molecular patterns and constitute the first line of defense against viral infections. Two types of IFNs are produced during the innate phase of the immune response: type I IFNs (13 subtypes of IFN-α, IFN-β, IFN-ε, IFN-k, and IFN-ω in humans) and type III IFNs (4 subtypes: IFN-λ1 or IL-29, IFN-λ2 or IL-28A, IFN-λ3 or IL-28B, and IFN-λ4 in humans) (1). These IFNs bind to specific receptors on target cells and initiate similar but non-redundant signaling pathways that lead to the expression of IFN-stimulated genes (ISGs) (2, 3). Proteins produced from those ISGs trigger an anti-viral state in the target cells that directly interfere with different steps of viral replication and indirectly modulate the host-immune response to virus infection (4–7). Due to their biological activity, IFNs have been studied or tested as therapeutic tools in the treatment of emerging and reemerging coronaviruses and other viral infections for which no approved drugs or vaccines are available (8–11).

The main difference between both IFN types is the location of their receptors. Type I IFNs recognize specific receptors that are ubiquitously expressed on the surface of all nucleated cells. Consequently, the clinical use of these molecules frequently causes side effects including fever, fatigue, and malaise mainly due to systemic proinflammation elicited on non-target cells (12, 13). Conversely, IFN-λ signals through the engagement of a heterodimeric receptor complex IFNLR1/IL10Rβ (IFNLR) whose expression is restricted to cells and tissues of epithelial origin, including epithelial cells of the respiratory and digestive tracts (1, 14). Due to the IFNLR location, IFN-λ constitutes the first line of defense controlling virus infection at the site of entry.

The COVID-19 pandemic has led to reconsider the use of available antivirals, and among them, IFNs. The use of IFNs is supported by the fact that SARS-CoV-2 induces a very weak endogenous expression of IFNs in infected cells (15–17) that may hamper the early innate immune response after infection. Hence, the use of exogenous IFNs, either for prophylaxis or early therapy to stimulate antiviral immunity, might be successful for treating COVID-19 (18, 19). In this context, IFN-λ has arisen as a promising candidate due to its localized activity on epithelial cells of the respiratory tract, which may reduce side effects and inflammation associated with the systemic action of type I IFNs.

One of the limitations of using human IFN-λ as a universal therapeutic molecule resides in the fact that it has several genetic variants (20, 21) that might have different stability and affinity in the interaction with the IFNLR. Engineering of IFN-λ to assess natural or *in silico* predicted mutations critical to maintaining the antiviral activity proved that the strength of the interaction of between IFN-λ and its receptor could modulate downstream functions (22–26). The strength of this interaction may modify the expression of the ISGs involved in the response to SARS-CoV-2 infection and even the virus receptor (ACE 2) on epithelial cells (27), promoting the reduced IFN signaling in infected cells. Seeking for an innovative high-performance low-cost IFN-λ for use in human health therapy, we developed a recombinant bovine IFN-λ expressed in HEK-293 cells (rbIFN-λ) hypothesizing that an enhanced binding capacity to the human heterodimeric receptor complex will improve its antiviral efficacy.

We have recently demonstrated that rbIFN-λ can activate the human Mx-promoter and that it has an effective antiviral activity *in vitro* against vesicular stomatitis virus (VSV), foot-and-mouth disease virus (FMDV), and bovine viral diarrhea virus (BVDV) (28). Moreover, treatment of calves with rbIFN-λ protected these animals from the disease caused by BVDV, downregulated the proinflammatory response, and promoted the development of the adaptive immune response (29). Here, we assessed for the first time the antiviral activity of this rbIFN-λ against SARS-CoV-2 *in vitro* and its safety on human immune cells. The affinity of bIFN-λ for the human receptor was also analyzed and compared to that of its human counterparts, following different *in silico* approaches.

## Materials and Methods

### Cells and Virus

HEK-293 cells were provided by the Argentinean Cell Bank at INTA and VERO-E6 cells by the Servicio Cultivos Celulares, INEI-ANLIS “Dr. Carlos G. Malbrán.” MDBK-t2 cells (30) were kindly provided by Dr. Bryan Charleston (The Pirbright Institute). Cell lines were maintained in Earle's Minimum Essential Medium (EMEM) containing 10% fetal bovine serum (FBS; Internegocios, Argentina), 2 mM L-glutamine, 1 mM sodium pyruvate, 1,500 mg/L sodium bicarbonate, 15 mM HEPES, and a commercial solution containing streptomycin (10 μg/ml), amphotericin B (0.025 μg/ml), and penicillin (10 UI/ml) at 37°C, 5% CO_2_. VERO cells cannot produce IFNs, but can respond to exogenous treatment (31).

Peripheral blood mononuclear cells (PBMCs) were purified from heparinized blood from two different volunteers using Histopaque® 1083 (Sigma-Aldrich, Thermo Fisher, DE USA) centrifuged at 1,000 × *g* for 30 min. A written informed consent was obtained from each peripheral blood donor, and procedures were in accordance with the 1964 Declaration of Helsinki and its later amendments and approved by National Ethics Committee of Buenos Aires Province through ACTA-2020-16644926-GBEBA-CECMSALGP.

A local strain of SARS-CoV-2 isolated from a clinical sample positive for COVID-19 in Buenos Aires, Argentina was used in this study. This strain was *in vitro* characterized by staff of the “Servicio Virosis Respiratorias INEI-ANLIS-Malbrán,” verifying its cytopathic effect (CPE) on VERO cells. Its whole genome was also sequenced (GISAID accession numbers EPI_ISL_420600). Viral stock was produced by infecting VERO cells and titrated following standard procedures. Briefly, serial 10-fold dilutions of the viral stock were plated in sextuplicate, and after 48–72 h of incubation at 37°C, the number of wells showing CPE was recorded. Viral titers were estimated using the Reed and Müench method and expressed in tissue culture infective dose 50% (TCID_50_)/ml (32).

### Recombinant Bovine IFN-λ

Details of sequence, cloning, and expression of the rbIFN-λ (bovine IFN-λ3, GenBank accession number HQ317919.1) have already been published (28). The batch used in this study was produced in HEK-293 cells and quantified in a reporter system using MDBK-t2 cells stably transfected with a construct that contains the human promoter of the MxA gene upstream of a reporter gene, chloramphenicol acetyltransferase enzyme (CAT) (30). Units of biologically active bovine rIFN-λ were measured by MxA-CAT ELISA as previously described (33) with some modifications. Briefly, MDBK-t2 cells were seeded into 12-well tissue culture plates at a density of 5 × 10^5^ cells/well. After 24 h of incubation at 37°C and 5% CO_2_, the culture medium was replaced with 500 μl of medium containing 250 μl of different dilutions of the rbIFN-λ preparation. Following a 24-h incubation, cells were washed with cold PBS 1 ×, lysed for 20 min in lysis buffer, and CAT expression was determined from the cell extracts by CAT ELISA kit (Roche Applied Sciences, IN, USA) following the manufacturer's instructions. Units of antiviral activity per milliliter of the samples were calculated from a standard curve using recombinant bovine IFN-α (from 0.3 to 5.0 IU/ml). The batch produced for this study contained 45 IU/ml of active rbIFN-λ. Recombinant human interferons (rhIFNs) α2b and β1a were kindly provided by Biosidus S.A. (Buenos Aires, Argentina) and contained 3 and 24 × 10^6^ IU/ml, respectively.

### *In silico* Analyses: Modeling and Docking

The sequences of bovine IFN-λ3 and human IFN-λ1, 2, 3, and 4 were retrieved from the GenBank and aligned for identity and similarity, identifying conserved critical regions (34).

Two different *in silico* approaches were used to predict the affinity of bIFN-λ for the human receptor. Using the crystallized ternary complex (hIFN-λ3/IFNLR) structure (PDB accession number 5T5W), a protein structural modeling was performed based on the amino acid sequences of human IFN-λ1, 2, and 3 and bovine IFN-λ3 (SWISS MODEL software; https://swissmodel.expasy.org/). This modeling allowed us to visualize the predicted interaction in the receptor pocket. Each IFN-λ variant was guided by distance restrictions between the Cα atom in contact between the ligand and the receptor and docked into either the structure of IFNLR1/IL10Rβ receptor or the IFNLR1 monomer alone (from PDB) using HDOCK server (http://hdock.phys.hust.edu.cn). After the docking was completed, we identified the 10 structures that yielded the lowest docking free energy for each IFN-λ and selected the one with the lowest root-mean-square deviation (RMSD) against the natural ligand. UCSF Chimera software was used to visualize the models. The dissociation constant (*K*_d_) and free Gibbs energy of binding (Δ*G*_bind_) were then estimated (Prodigy server https://bianca.science.uu.nl//prodigy/).

Using the hIFN-λ3/IFNLR complex structure, the interface residues between hIFN-λ3 and each subunit of the heterodimeric receptor were determined using PDB SUM database (35). This crystallographic structure (5T5W) is already an IFN-λ3 mutant (mut-hIFN-λ3) conceived to improve the binding affinity for its receptor (Mendoza 2017). Mutation on the interface residues of the mut-hIFN-λ3 was incorporated using FoldX software (Schymkowitz 2005), creating new variants with replaced interface residues present in hIFN-λ3 or bIFN-λ3, and the Δ*G*_bind_ of the interaction of the ligand–receptor complex was estimated. Mutant structures were visualized using VMD software (36).

### Viability Assessment

The metabolic activity of VERO cells and PBMCs pretreated with 4.5, 9, and 18 IU/ml of rbIFN-λ was measured with TACS® XTT Cell proliferation Assay Kit (TREVIGEN, Gaithersburg, MD, USA) according to the manufacturer's instruction and as previously reported (37). OD values for mock-treated cells were computed as reference of viable cells. Control dead cells were obtained by performing an osmotic shock, incubating the cells overnight (ON) with PBS. Percentage of living cells was referred to values of untreated control wells. Samples were run in triplicate.

PBMCs were also stained with a LIVE/DEAD™ Fixable Dead Cell Stain Kit (Thermo Fisher), according to the manufacturer's recommendations. Fluorescence intensity was determined by FACS analysis at 665 nm (BD Biosciences FACSCalibur™), and results were analyzed using a specific software (FlowJo V10; BD, OR USA).

### Cytokine Responses

Heparinized whole blood samples from two different donors were incubated at 37°C and 5% CO_2_ with LPS (20 ng/ml, Sigma Aldrich–Thermo Fisher); rbIFN-λ (5 IU/ml) or both rbIFN-λ and LPS were mock treated. After 24 h of incubation, plasma samples were separated by centrifugation (1,200 × *g*, 10 min) and tested for IL-6 and IL-10 production by a chemiluminescent assay at a private clinical laboratory (IACA Laboratorios, Argentina).

### Antiviral Activity Against SARS-CoV-2

VERO cells were seeded into 96-well tissue culture plates (1.5 × 10^4^ cells per well) 24 h prior to treatment with serial dilutions (0.0175 to 18 IU/ml) of rbIFN-λ and recombinant human IFN-α2b and IFN-β1a (rIFN-α and rIFN-β, respectively), kindly provided by Biosidus SA. (Argentina), as control treatments. After an ON incubation, the supernatants were removed and cells were infected with SARS-CoV-2 at a MOI of 0.5 in infection medium (as it was previously described but containing only 2% FBS) for 1 h. Medium containing the inoculum was removed and replaced with 200 μl per well of fresh medium (2% FBS) supplemented with the corresponding rIFN at the indicated concentrations or medium alone. Plates were incubated for 48 h, when infected cell control wells showed CPE. At this time point, cell supernatants were collected, pelleted for 10 min at 6,000 × *g* to remove debris, and then transferred to sterile collection tubes for RNA extraction. The cell monolayers were stained with crystal violet, and the resulting OD read at 575 nm in a microplate reader (Synergy H1, BioTek, USA). These results were used to calculate the corresponding IFN concentration that provided 50% of protection to the infection of the cells in culture (inhibitory concentration 50, IC_50_). Triplicate wells containing IFN-treated non-infected cells were run in parallel as toxicity controls in every experiment.

### Detection of SARS-CoV-2 Using a TaqMan qRT-PCR Assay

The antiviral activity of the rbIFN-λ in VERO cells with the SARS-CoV-2 was also assessed by detecting viral genome in cell culture supernatants through an optimized qRT-PCR assay. Briefly, 140 μl of cell culture supernatants seeded in quadruplicates was processed to extract total RNA using the QIAamp Viral RNA Mini Kit (Qiagen, Germany). Reverse transcription and amplification of SARS-CoV-2 E-gene were performed using the Lightmix Modular SARS-CoV (COVID-19) (TIB MOLBIOL-Roche, Switzerland) and the SuperscriptTM III Platinum OneStep qRT-PCR kits (Invitrogen, Thermo Fisher) and run on an ABI 7500 Real-Time PCR System (Applied BioSystems, Thermo Fisher) following standard procedures. Reverse transcription was done at 50°C for 10 min, followed by a polymerase activation and target denaturation step at 95°C for 10 min, and PCR amplification was run at 95°C for 15 s and 58°C for 35 s (45 cycles). All reactions were performed in a final volume of 25 μl, containing 5 μl of total RNA. A reference curve built upon serial dilutions ranging from 6 × 10^−1^ to 6 × 10^6^ copies/μl was used to calculate the number of genome copies in each sample, using standards provided by the Pan American Health Organization (SARS-like Wuhan, Iv-RNA E gene standard 1 × 10^8^ copies/μl and SARS-like Wuhan, Iv-RNA RdRP gene standard, 1 × 10^8^ copies/μl). The reduction of the number of SARS-CoV-2 genome copies was also used to estimate IC_50_, as described for the cell monolayer staining method.

As for the previous section, all experiments involving infective SARS-CoV-2 were performed by the staff of the “Servicio Virosis Respiratorias INEI–ANLIS Dr. Carlos G. Malbrán” at the ANLIS “Dr. Carlos G. Malbrán” BSL-3 facilities.

### Statistical Analysis

The standard curve used to estimate viral RNA quantities was run in triplicate and analyzed using GraphPad Prism 9. Results obtained for antiviral activity against the different IFN concentrations were compared using one-way ANOVA Kruskal–Wallis test, followed by Dunn's multiple comparison test. Normal distribution of these values was previously confirmed using the D'Agostino-Pearson normality test (GraphPad Prism 9). The confidence interval used was 95% or 99% depending on the experiment.

## Results

### Interaction of rbIFN-λ With Human Receptors

There are four human IFN-λ coding sequences clustered at chromosome 9: IFN-λ1 (IL29), IFN-λ2 (IL28A), IFN-λ3 (IL28B), and IFN-λ4. Identity between the amino acid sequences of human IFN-λ was first analyzed (Table 1A). The highest degree of identity was found between human IFN-λ3 and IFN-λ2 (96%), followed by the comparison to IFN-λ1 (80%). Identity between human IFN-λ1 and λ2 was 71%, while IFN-λ4 was very different to all the other human IFN-λ (identities <30%). We then compared human IFN-λ1 to IFN-λ4 with the sequence of the rbIFN-λ. Interestingly, the percentage of identical residues were equivalent when compared to human IFN-λ1, 2, and 3 sequences (between 64 and 68%) as well as the similarity that ranged between 74 and 75% (Table 1B). As expected from the comparison among human IFN-λ, both the identity and similarity between the rbIFN-λ and hIFN-λ4 were much lower (30 and 43%, respectively). Due to its differences with the other IFN-λ variants under study (both human and bovine), the hIFN-λ4 was excluded from further analysis.

**Table 1:** Analysis of bovine and human IFN-λ sequences.

	**hIFN-λ2**	**hIFN-λ3**	**hIFN-λ4**
**A**. Identity between human IFN-λ			
hIFN-λ1	139/196 (71%)	153/189 (80%)	54/190 (28%)
hIFN-λ2	–	188/196 (96%)	44/171 (26%)
hIFN-λ3	–	–	45/175 (26%)
	**Identities**	**Similarities**	**Expect**
**B**. Identity and similarity between bovine and human IFN-λ			
hIFN-λ1	111/174 (64%)	131/174 (75%)	5e−74
hIFN-λ2	131/198 (66%)	148/198 (74%)	8e−84
hIFN-λ3	134/198 (68%)	149/198 (75%)	2e−86
hIFN-λ4	48/160 (30%)	70/160 (43%)	4e−11

***(A)** Identity of the amino acid sequences between human IFN-λ1 and human IFN-λ4. **(B)** Identities, positives (similarity), and E-values of the alignments between human IFN-λ1 and human IFN-λ4 and the rbIFN-λ sequence*.

To the best of our knowledge, the potential antiviral activity of bovine IFN-λ on human cells has never been assessed. An *in silico* analysis was first performed to predict the tridimensional structure of the interaction between the bIFN-λ and the human receptor (IFNLR). A protein structure was modeled using the amino acid sequences of the bIFN-λ and the hIFN-λ1, 2, and 3, and the crystal structure of the human IFN-λ3/IFNLR complex was used as template. With these models, PDB files were generated and run in a docking software to visualize the predicted interaction in the receptor pocket, where IFN-λ binds to trigger the JAK/STAT pathway. Both human and bovine IFN-λ exhibited similar secondary and tertiary structures, thus suggesting that they may interact similarly with the IFNLR (Supplementary File 1).

The interaction between IFNLR and each modeled IFN-λ was studied in terms of stability through a docking assessment. The free Δ*G*_bind_ and the *K*_d_ for the best model generated by the docking procedure were computationally estimated. As it is shown in Table 2A, the bovine IFN-λ3/IFNLR prediction yielded the lowest Δ*G*_bind_ and *K*_d_ values, suggesting a more stable interaction between the bIFN-λ and the IFNLR compared to the human IFN-λ. The highest binding stability of bovine IFN-λ was also observed in the interaction with the monomer IFN-λR1 (data not shown).

**Table 2:** Interaction with the human IFNLR.

**Docking**	**Δ*G*_**bind**_ (kcal/mol)**	***K*_**d**_**
**A**. Predicted stability values from IFN-λ/IFNLR interaction		
hIFN-λ1/IFNLR	−12.3	9.30E−10
hIFN-λ2/IFNLR	−12.4	8.30E−10
hIFN-λ3/IFNLR	−13.1	2.30E−10
bIFN-λ/IFNLR	−13.9	6.90E−11
**ID**	**Δ*****G***_**bind**_ **(kcal/mol)**	
**B**. Stability values from *in silico* mutagenesis		
WT hIFN-λ3/IFNLR	−36.9141	
bIFN-λ/IFNLR	−38.6697	
mut-hIFN-λ3/IFNLR	−39.0803	

***(A)** The interaction between IFNLR and each modeled IFN-λ was studied in terms of stability through a docking assessment. The free Gibbs energy of binding (ΔG_bind_) and the dissociation constant (K_d_) were computationally estimated. **(B)** An in silico mutagenesis analysis was performed using FoldX software by replacing the interface residues present in both the wild-type hIFN-λ3 and in bIFN-λ. The free Gibbs energy of binding (ΔG_bind_) of the interaction of the ligand–receptor complex is depicted. hIFN-λ1–3: human IFN-λ1 to 3; bIFN-λ: bovine IFN-λ; WT hIFN-λ3: wild-type human IFN-λ3; mut-hIFN-λ3: mutant human IFN-λ3; IFNLR: human IFN-λ receptor*.

**Figure 1:**
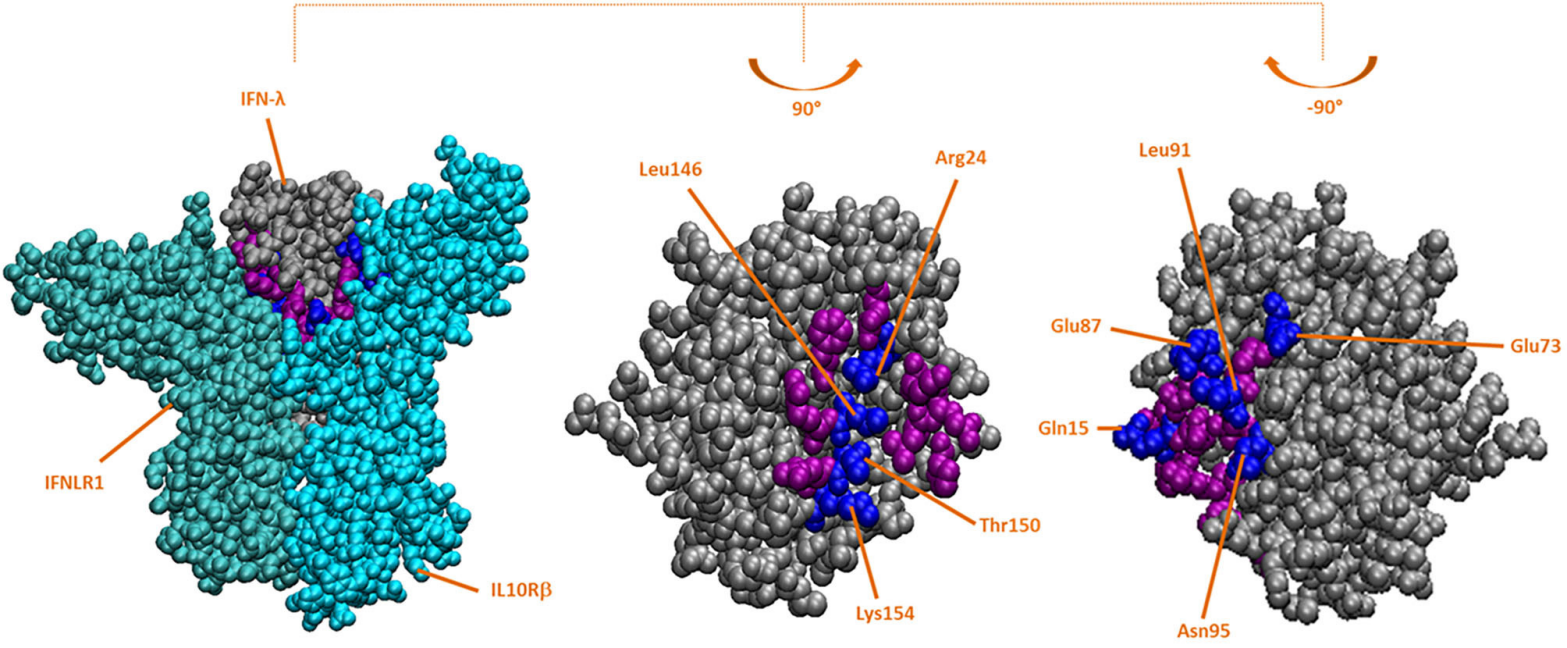
Crystallographic structure of hIFN-λ and hIFN-λ/IFNLR *in silico*-mutated complex. Based on the crystallographic structure of the hIFN-λ3/IFNLR complex (PDB: 5T5W), IFN-λ residues in the interface with IFNLR were replaced with those present in the bovine interface using FoldX software. All residues in the IFN-λ interface are colored purple and blue, and mutated residues (in blue) are indicated. Left: mutated structure of the hIFN-λ/IFNLR1/IL10Rβ complex; side faces of mutated hIFN-λ interacting with IFNLR1 (center) and IL10Rβ (right).

Based on the crystallographic structure of the hIFN-λ3/IFNLR complex, interface residues were identified (Figure 1). Some of them had been mutagenized previously to obtain the crystal structure (25). The impact of these residues present in both wild-type human and bovine IFN-λ3 on the stability of the interaction with IFNLR was assessed by an *in silico* mutagenesis analysis. These interface residues present in the mut-hIFN-λ3 were replaced by the bovine and wild-type IFN-λ3 amino acids and fitted within the hIFN-λ3/IFNLR structure. The Δ*G*_bind_ of the interaction was determined (Table 2B). The mutations performed on the PDB structure of mut-hIFN-λ3 were H95N, R15Q, H91L, D87E, and D73E (Figure 1, center), and K24R, F146L, A150T, and N154 (Figure 1, right), based on the amino acid residues present in bIFN-λ. According to Δ*G*_bind_ values, bovine IFN-λ3 showed higher affinity for the IFNLR than human wild-type IFN-λ3, and the interface amino acids of the bovine sequence may be responsible for this increased interaction efficacy.

### Safety of rbIFN-λ

In order to be used as a human therapeutic agent, rbIFN-λ must be safe for human cells and unable to upregulate proinflammatory cytokines in immune cells.

In a first experiment, PBMCs from two different healthy donors were incubated ON with 4.5, 9, or 18 IU/ml of rbIFN-λ, stained with a specific marker to differentiate between live and dead cells and analyzed by FACS (Supplementary File 2). Viable and dead cells were quantified as a whole and by gating events according to their size and granularity to identify lymphocytes, granulocytes, and monocytes. No differences were recorded in the number of dead and live cells associated to the increasing concentrations of rbIFN-λ assessed. Mortality rate yielded values below 1% for all samples, even when 18 IU/ml of rbIFN-λ were used, and not different to those found in the mock-treated cells (Table 3A). Likewise, no changes in cell size or granularity were found after rbIFN-λ treatment, and the percentage of granulocytes, monocytes, and lymphocytes were almost identical between mock and rbIFN-λ treatments (Table 3B).

**Table 3:** Bovine rIFN-λ is safe for human immune cells.

		**Treatment**			
		**Mock**	**rbIFN-λ (4.5 IU/ml)**	**rbIFN-λ (9 IU/ml)**	**rbIFN-λ (18 IU/ml)**
**A**. Percentage of dead cells					
DONOR 1		0.82 ± 0.11	1.01 ± 0.99	0.74 ± 0.37	0.72 ± 0.38
DONOR 2		0.58 ± 0.29	0.41 ± 0.08	0.98 ± 0.55	0.90 ± 0.42
**B**. Percentage of total cells					
Donor 1	Granulocytes	24.5 ± 0.92	26.4 ± 1.62	26.4 ± 0.62	24.0 ± 1.93
	Monocytes	4.66 ± 0.15	5.56 ± 0.51	5.32 ± 0.64	4.82 ± 0.10
	Lymphocytes	58.4 ± 1.45	53.0 ± 1.07	56.0 ± 2.83	57.7 ± 2.06
Donor 2	Granulocytes	43.9 ± 1.9	45.5 ± 1.6	42.3 ± 1.9	41.5 ± 3.6
	Monocytes	3.70 ± 0.99	3.57 ± 1.2	4.16 ± 1.1	2.86 ± 0.8
	Lymphocytes	44.8 ± 1.70	41.9 ± 0.96	41.7 ± 4.8	42.4 ± 5.57

*Leucocytes were purified from heparinized blood from two healthy volunteers and treated (or mock-treated) with increasing concentrations of bovine rIFN-λ (4.5; 9; and 18 IU/ml). After ON incubation, cells were stained with LIVE/DEAD™ Fixable Dead Cell Stain Kit and analyzed by FACS. **(A)** Lymphocytes were gated based on the morphological criteria (SSC vs. FSC cytogram), and the percentage of dead cells after each treatment was estimated. **(B)** Lymphocytes, monocytes, and granulocytes were gated based on the morphological criteria (SSC vs. FSC cytogram), and the percentage of total cells within each cell type was estimated and compared between treatments. Mean values ± SD from triplicate samples are depicted for each treatment*.

**Figure 2:**
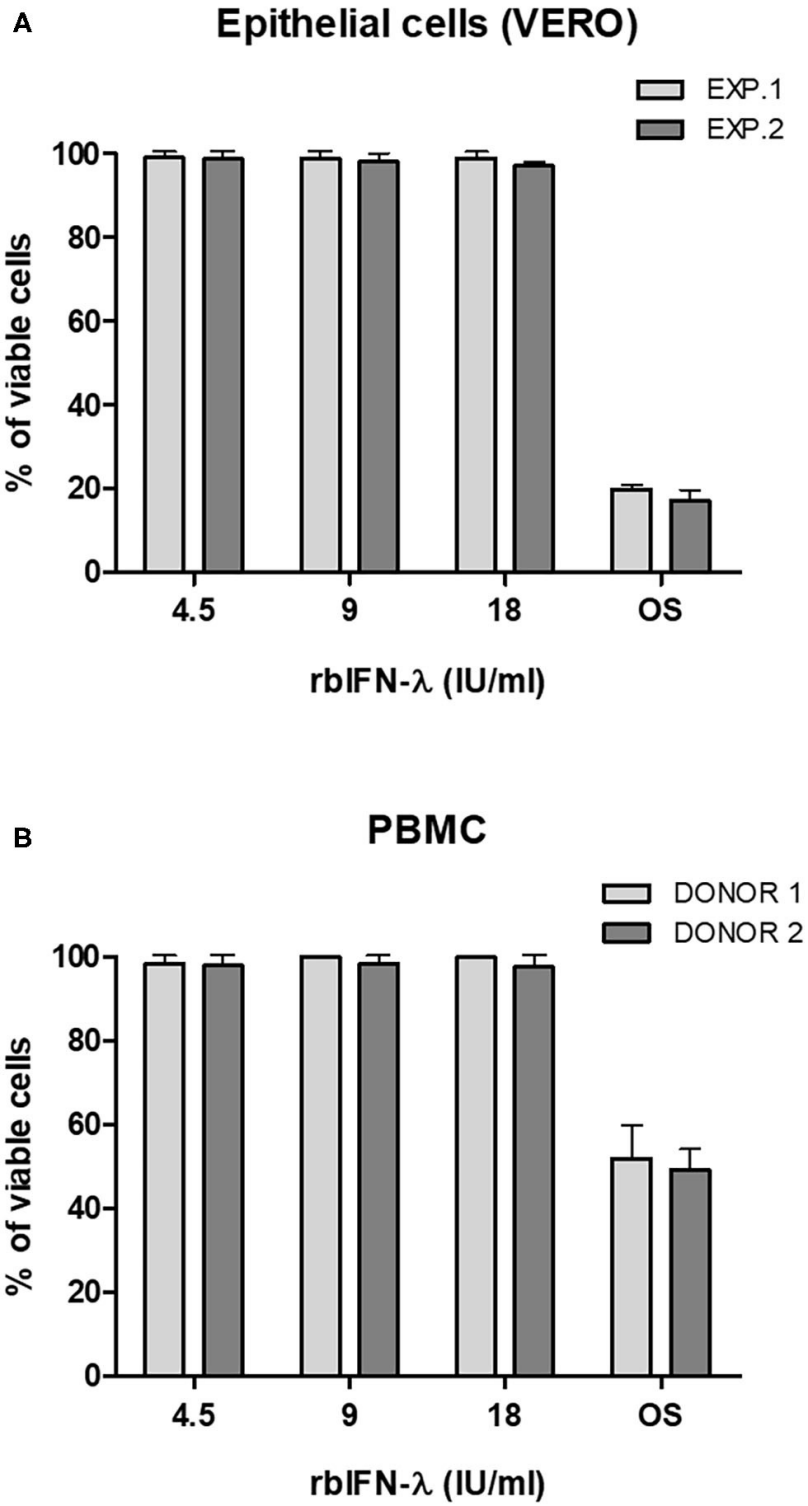
Effect of the bovine rIFN-λ on the viability of VERO cells and human PBMCs. VERO cells **(A)** and PBMCs **(B)** were treated with 4.5, 9, and 18 IU/ml of recombinant bovine IFN-λ (rbIFN-λ) for 18 h and their capacity to reduce XTT was assessed. Control cell samples were also incubated ON with PBS to induce an osmotic shock (OS). Percentage of living cells was referred to values of mock-treated controls. Mean values ± SD from triplicate samples are depicted for each dilution for each experiment (EXP.) or human donor.

Safety of rbIFN-λ was then assessed by measuring the metabolic activity of VERO cells (Figure 2A) and human PBMCs (Figure 2B) after an ON incubation with the same concentrations of rbIFN-λ used in the previous experiment. A colorimetric assay was used, and the percentage of living cells was referred to values of mock-treated cells. No changes in the viability of any of these cells were observed even at the highest rbIFN-λ concentration assayed.

Whole blood samples from the same donors were also treated ON with 18 IU/ml of rbIFN-λ, 20 ng/ml of LPS, and a mixture of rbIFN-λ and LPS. The following day, IL-6 and IL-10 levels were measured in stimulated plasma. Both IL-6 and IL-10 levels were lower in rbIFN-λ-treated PBMCs compared to LPS-treated samples. Interestingly, detection of IL-10 was reduced when LPS and rbIFN-λ were used together, compared to LPS alone (Table 4).

**Table 4:** Effect of the bovine rIFN-λ on the induction of inflammatory responses in human immune cells.

		**Treatment**			
		**Mock**	**LPS**	**rbIFN-λ**	**LPS + rbIFN-λ**
Donor 1	IL-6	<2	>10,000	1,650	>10,000
	IL-10	<5	>1,000	22.2	391
Donor 2	IL-6	<2	>10,000	270	>10,000
	IL-10	<5	757	<5	117

*Whole blood samples were stimulated ON with LPS (20 ng/ml), rbIFN-λ (18 IU/ml), a combination of both, or mock-treated with dilution buffer (PBS). IL-6 and IL-10 were quantified by a chemiluminescent assay, and values were expressed in pg/ml*.

### Activity of rbIFN-λ Against SARS-CoV-2

Activity of rbIFN-λ against SARS-CoV-2 was assessed in three independent experiments using samples run in quadruplicates. VERO cells were incubated ON with rbIFN-λ, human rIFN-α, or rIFN-β and infected with an Argentinean isolate of SARS-CoV-2. Mock-infected cells and IFN-treated non-infected wells were used as controls. Cultures were examined for CPE at 24 h and 48 h, when supernatants were recovered for quantitation of SARS-CoV-2 genome copies, and cells were fixed and stained for colorimetric assessment.

**Figure 3:**
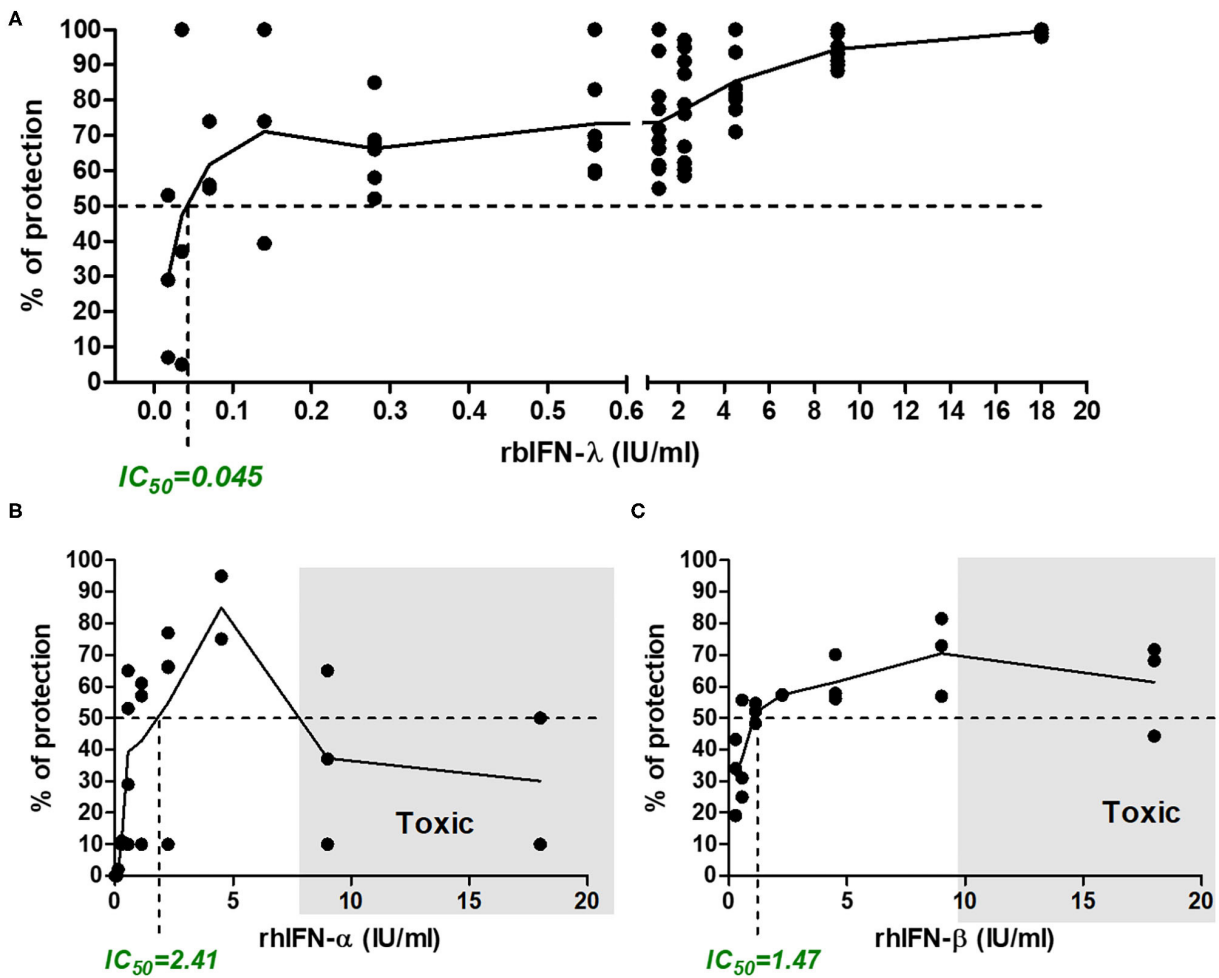
Antiviral activity of bovine rIFN-λ in VERO cells infected with SARS-CoV-2. VERO cells were treated with serial dilutions of bovine rIFN-λ **(A)**, human rIFN-α **(B)**, or human rIFN-β **(C)**. After an ON incubation, cells were infected with SARS-CoV-2 (MOI = 0.5) and incubated for 48 h, when cytopathic effect was detected in untreated cells. Antiviral activity was estimated by staining the cell monolayers with crystal violet and reading the resulting OD at 575 nm. Percentage of protection was referred to values of mock-treated and infected cells. Toxicity controls were run in parallel in every experiment on treated uninfected cells. The inhibitory concentration 50% (IC_50_, concentration required to inhibit SARS-CoV-2 infection in 50% of the replicates analyzed) is indicated in each chart. Shaded areas depict the toxic concentration range, defined as the concentration of IFN that induces a loss of viability in uninfected cells (a cytopathic-like effect is observed microscopically in IFN-treated but not infected wells, with an increasing number of dead cells, which is related to the low-OD 575-nm values).

Incubation with rbIFN-λ did not produce any adverse effect in VERO cells even at the highest concentration (18 IU/ml). On the contrary, incubation with high concentrations of human rIFN-α and rIFN-β was toxic for the cells in culture. About 60% of the cells were killed by rhIFN-α and 25% were killed by rhIFN-β used at a concentration of 9 IU/ml (Figures 3B,C).

As shown in Figure 3A, the rbIFN-λ had a strong antiviral activity against SARS-CoV-2. The estimation of rbIFN-λ IC_50_ with the colorimetric assay was 0.045 IU/ml, 53 times lower than that of human IFN-α and almost 33 times lower than rhIFN-β. These results were consistent with CPE observation (Figure 3 and Supplementary File 3).

**Figure 4:**
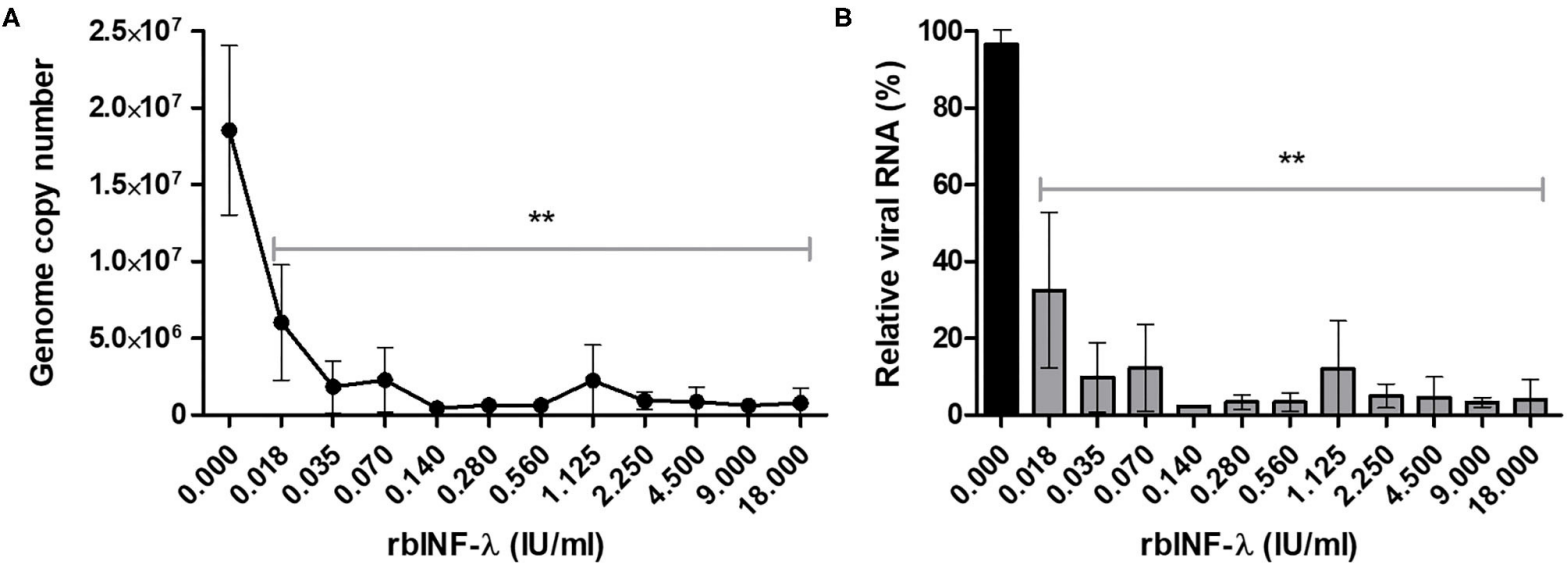
Detection of SARS-CoV-2 genome. VERO cells were infected with serial dilutions of bovine rIFN-λ and SARS-CoV-2 RNA from culture supernatant samples and were quantified by qRT-PCR. Results are expressed as viral RNA copy number per milliliter **(A)** and percentage of relative viral RNA from mock-treated and infected cells **(B)**. Mean values ± SD from quadruplicate samples are depicted for each rbIFN-λ dilution. **Values significantly lower than those measured in the mock-treated and infected cells (*p* < 0.01).

RT-qPCR results also showed that all the concentrations of the rbIFN-λ tested caused a reduction of viral RNA copy number that was significant with respect to untreated infected cells (*p* < 0.01; Figure 4). Moreover, viral genome copy numbers were drastically reduced by 2 log_10_ units of magnitude at a concentration of 0.1 IU/ml and were almost undetectable by the assay at higher concentrations of rbIFN-λ (Figure 4A). The incubation with rbIFN-λ at concentrations as low as 0.02 IU/ml reduced the yield of viral RNA produced by mock-treated infected cells to < 10% (Figure 4B). We estimated that 0.008 IU/ml of rbIFN-λ might reduce the copy number of genomic viral RNA produced by untreated infected cells by 50%. These results demonstrate that rbIFN-λ is a potent inhibitor of the SARS-CoV-2 clinical Argentinean isolate.

## Discussion

Administration of IFNs can be used for prophylaxis and early therapy of COVID-19 compensating the weak IFN response in the first stages of human SARS-CoV-2 infection (38, 39). IFN-λ has several advantages compared to type-I IFNs and is already under clinical trials (40). In this study, we assessed the efficacy of a recombinant bovine IFN-λ against SARS-CoV-2. We confirmed the *in vitro* safety and enhanced efficacy of this IFN preventing SARS-CoV-2 infection in VERO cells at concentrations significantly lower than those required for recombinant human IFN-α and -β. To the best of our knowledge, this is the first time a bovine IFN has been proposed as a human biotherapeutic.

The use of bovine IFN-λ for human use is supported by its capability of activating the human Mx promoter (28); its high similarity with human IFN-λ1, 2, and 3; and a predicted higher affinity for the human IFNLR1/IL10Rβ heterodimeric receptor, at least in terms of free energy and dissociation constant. It is important to consider that due to the limited available crystallized structures, we modeled the rbIFN-λ using the human IFN-λ/IFNLR complex as template. Even though there are high similarities in the linear amino acid alignment, the predictive modeling is limited by the backbone conformation of the template and adjustments of side-chain stereochemistry-based differences with the model, possibly concealing real structural differences between the human and bovine proteins. Notwithstanding these limitations, our *in silico* analysis revealed that the enhanced affinity was mainly related to discrete amino acid substitutions. Interestingly, some of these positions had been mutagenized before to obtain a stable interaction for the crystallographic structure assessment (25). These observations support the idea that affinity of human IFN-λ for its receptor may be improved and that a heterologous IFN-λ (such as this rbIFN-λ) could have a better affinity for the human IFNLR. Improving IFN-λ affinity might increase the downstream signaling and improve the activation of the ISGs, reducing the effective dose needed for therapeutic use.

Safety of the rbIFN-λ in human immune cells was confirmed by using different viability assays. An ON incubation of rbIFN-λ in doses as high as 18 IU/ml with human PBMCs did not affect metabolic activity, viability, size, or granularity of these cells. No cytotoxicity signs were found for VERO cells even at higher concentrations than those that killed these cells when treated with recombinant human IFN-α and -β.

The rbIFN-λ induced a cytokine pattern on human PBMCs similar to that reported for human IFN-λ, upregulating IL-6 and inducing low levels of IL-10 (41), thus confirming a comparable immune activity of rbIFN-λ in human immune cells. This cytokine profile is expected to activate the innate immunity at the site of viral infection and promote the development of the acquired immunity. Our results show that the co-treatment of PBMCs with rbIFN-λ and LPS reduced IL-10 levels compared to LPS alone, which can modulate inflammation produced by bacterial infections (42, 43). The limited proinflammatory effect is one of the most relevant advantages of IFN-λ compared to type 1 IFNs (44), particularly for treating COVID-19, as inflammation has been associated with the development of severe disease. However, the direct effect of IFN-λ on COVID-19 progression remains unclear and the responsiveness of human immune cells to IFN-λ is still being analyzed (22). In this scenario and with IFN-λ being quite recently discovered (45, 46), more work is needed to elucidate the role of this cytokine and the timing of its application to prevent or reduce the progression of COVID-19.

Several studies show that type I and type III IFNs are effective in reducing SARS-CoV-2 replication in VERO cells (18, 19, 47).

Lokugamage et al. recently demonstrated that a pretreatment of VERO cells with 1,000 IU/ml of human IFN-α caused a 2-log_10_ drop in viral titer at 48 dpi as compared to control untreated cells (47). We found the same result but using 0.14 IU/ml of rbIFN-λ. Another study from Mantlo et al. (19) estimated the IC_50_ of IFN-α and IFN-β treatment of VERO cells before SARS-CoV-2 infection to be 1.35 IU/ml and 0.76 IU/ml, respectively. These values are similar to those estimated here for type I human IFNs and over 30 times higher than the one computed in this study for the rbIFN-λ. Although comparisons are difficult due to the use of different IFN-quantitation methods and the various readouts used for the infection assessments, bovine IFN-λ seems to be more efficient than human type I IFNs to prevent SARS-CoV-2 infection *in vitro*.

Felgenhauer et al. showed that 10 ng/ml of rhIFN-λ significantly reduced SARS-CoV-2 titers in VERO cells. Using our production method, we estimate that 1 IU corresponds to 10 ng of rbIFN-λ, meaning that 0.17 ng of our rbIFN-λ (0.0175 IU/ml) would be sufficient to reducing SARS-CoV-2 replication in VERO cells. These results suggest that about 50 times lower concentrations of bovine IFN-λ are required to achieve a similar reduction rate than that achieved by human IFN-λ (18). These estimations will be confirmed by a side-by-side assay using recombinant human IFN-λ in future experiments.

Our results demonstrate that rbIFN-λ is more efficient than two recombinant human type I (α- and β-) IFNs in impeding SARS-CoV-2 infection in VERO cells. This evidence, together with its low *in vitro* toxicity, the biological functions of the type III IFNs, its high-sequence identity with human counterparts, and its predicted enhanced binding capacity to the human IFNLR, supports further evaluations of the rbIFN-λ as a potential biotherapeutic compound for COVID-19 that could be produced at affordable costs. Moreover, this strategy could be tested against other respiratory viral infections that may emerge.

We have already proved the versatility of producing active rbIFN-λ in HEK-293 cells, *Escherichia coli*, or by using a recombinant baculovirus in insect cells (data not shown). We envision a formulation that can be administered locally through an inhaler (puffer) or using a nebulizer either early after infection or as a preventive measure, two options that have been successfully applied for human IFNs (39). A simple administration method and the expected low cost of this antiviral are paramount issues for low–middle-income countries (LMIC) like ours, with significant percentages of the population with limited access to health services and lacking even basic healthcare needs. These therapeutic alternatives may also be relevant in a middle-term scenario for LMIC where COVID-19 vaccines will be available on limited grounds and firstly used in the high-risk population, reinforcing the need for a low-cost therapeutic to counteract future waves of SARS-CoV-2 infection.

## Data Availability Statement

The raw data supporting the conclusions of this article will be made available by the authors upon request.

## Ethics Statement

The studies involving human participants were reviewed and approved by the committee approved the use of human blood samples for our *in vitro* experiments. Our protocol was approved by the Comité de Ética Central of the Buenos Aires Province Government, Exp 2919-2182-2020, resolution number ACTA-2020-16644926-GDEBA-CECMSALGP dated August 12th, 2020. The patients/participants provided their written informed consent to participate in this study.

## Author Contributions

NC and FM developed the IFN-λ batch, carried out most of the experiments, and helped in data analyses. EBe did all the experiments with live virus supervised by EBa. LB and JI carried out all the bioinformatics evaluations. IS performed the flow cytometry analyses. CT and LB analyzed all relevant literature and helped in drafting the manuscript. AC designed and directed the study, analyzed the data, and wrote the manuscript. All authors read and approved the final manuscript.

## Conflict of Interest

The authors declare that the research was conducted in the absence of any commercial or financial relationships that could be construed as a potential conflict of interest.
