# Supplementary material for: Bovine Interferon Lambda Is a Potent Antiviral Against SARS-CoV-2 Infection in vitro
Source: Front Vet Sci. 2020 Nov 6;7:603622. doi: 10.3389/fvets.2020.603622 (PMC7677234; doi:10.3389/fvets.2020.603622)
Supplement: Supplementary file 1 [file Presentation_1.PPTX]

## Slide 1
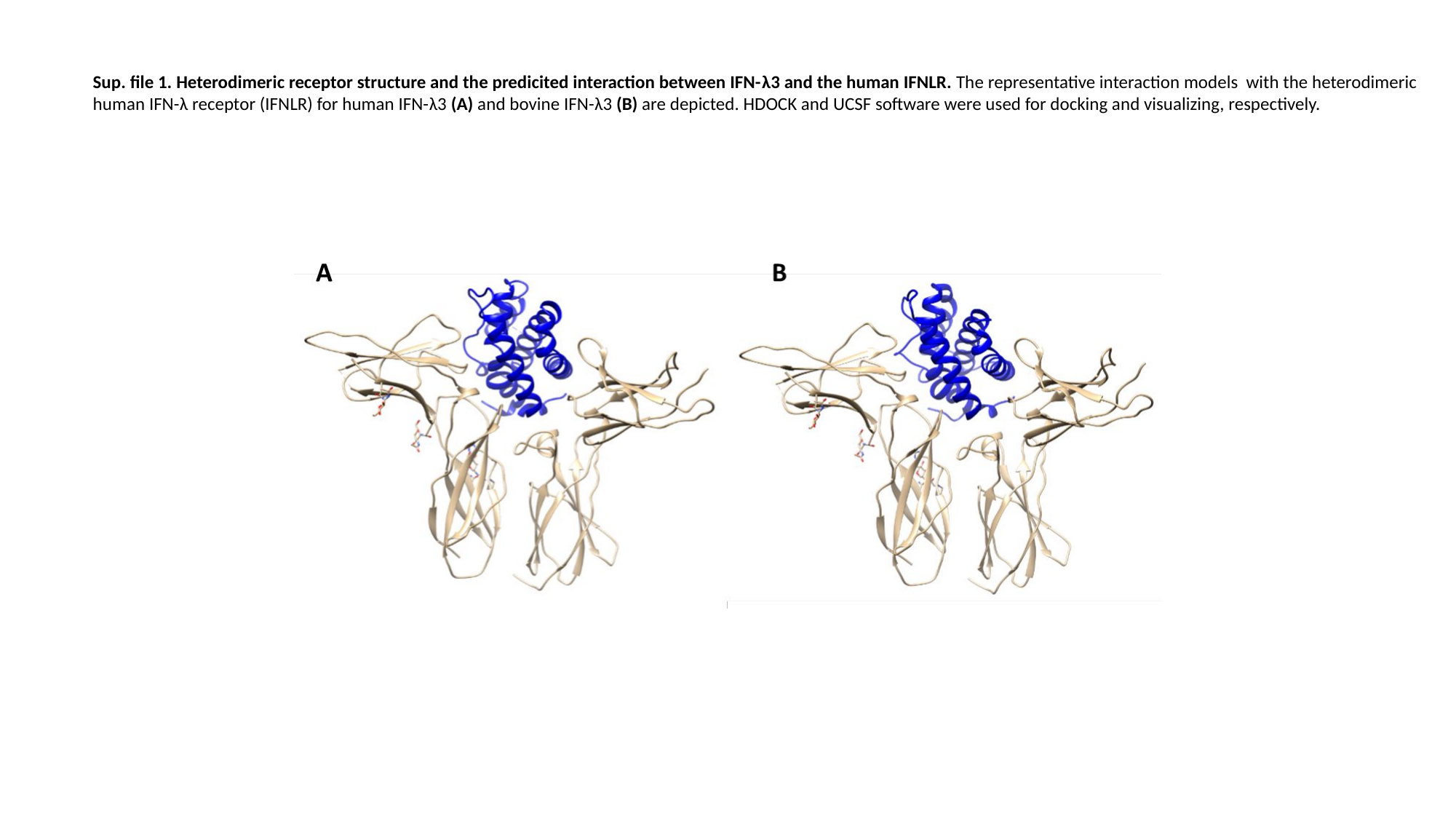

Sup. file 1. Heterodimeric receptor structure and the predicited interaction between IFN-λ3 and the human IFNLR. The representative interaction models with the heterodimeric human IFN-λ receptor (IFNLR) for human IFN-λ3 (A) and bovine IFN-λ3 (B) are depicted. HDOCK and UCSF software were used for docking and visualizing, respectively.
