# Supplementary material for: Bovine Interferon Lambda Is a Potent Antiviral Against SARS-CoV-2 Infection in vitro
Source: Front Vet Sci. 2020 Nov 6;7:603622. doi: 10.3389/fvets.2020.603622 (PMC7677234; doi:10.3389/fvets.2020.603622)
Supplement: Supplementary file 2 [file Presentation_2.PPTX]

## Slide 1
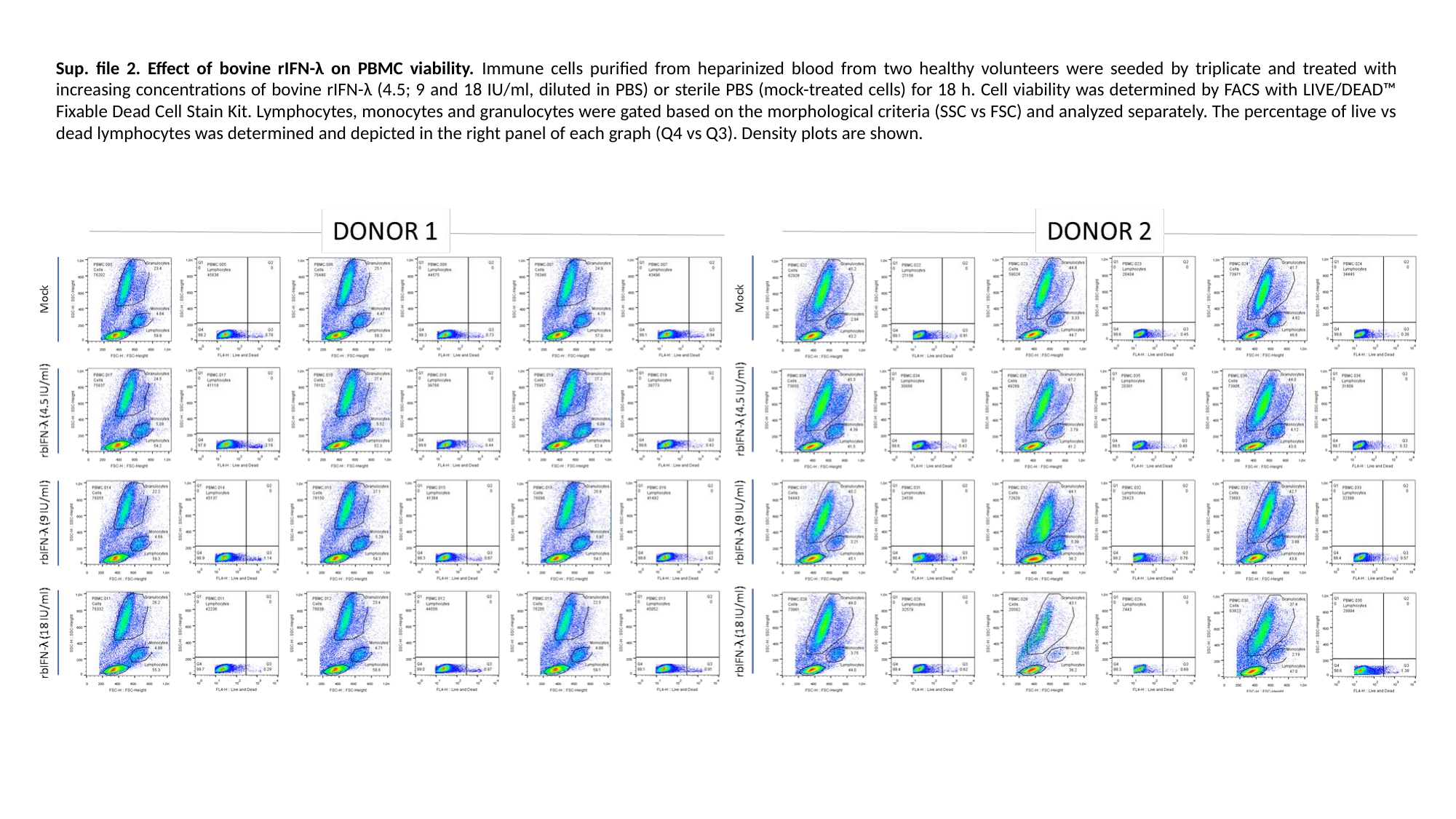

Sup. file 2. Effect of bovine rIFN-λ on PBMC viability. Immune cells purified from heparinized blood from two healthy volunteers were seeded by triplicate and treated with increasing concentrations of bovine rIFN-λ (4.5; 9 and 18 IU/ml, diluted in PBS) or sterile PBS (mock-treated cells) for 18 h. Cell viability was determined by FACS with LIVE/DEAD™ Fixable Dead Cell Stain Kit. Lymphocytes, monocytes and granulocytes were gated based on the morphological criteria (SSC vs FSC) and analyzed separately. The percentage of live vs dead lymphocytes was determined and depicted in the right panel of each graph (Q4 vs Q3). Density plots are shown.
