# Supplementary material for: Bovine Interferon Lambda Is a Potent Antiviral Against SARS-CoV-2 Infection in vitro
Source: Front Vet Sci. 2020 Nov 6;7:603622. doi: 10.3389/fvets.2020.603622 (PMC7677234; doi:10.3389/fvets.2020.603622)
Supplement: Supplementary file 3 [file Presentation_3.PPTX]

## Slide 1
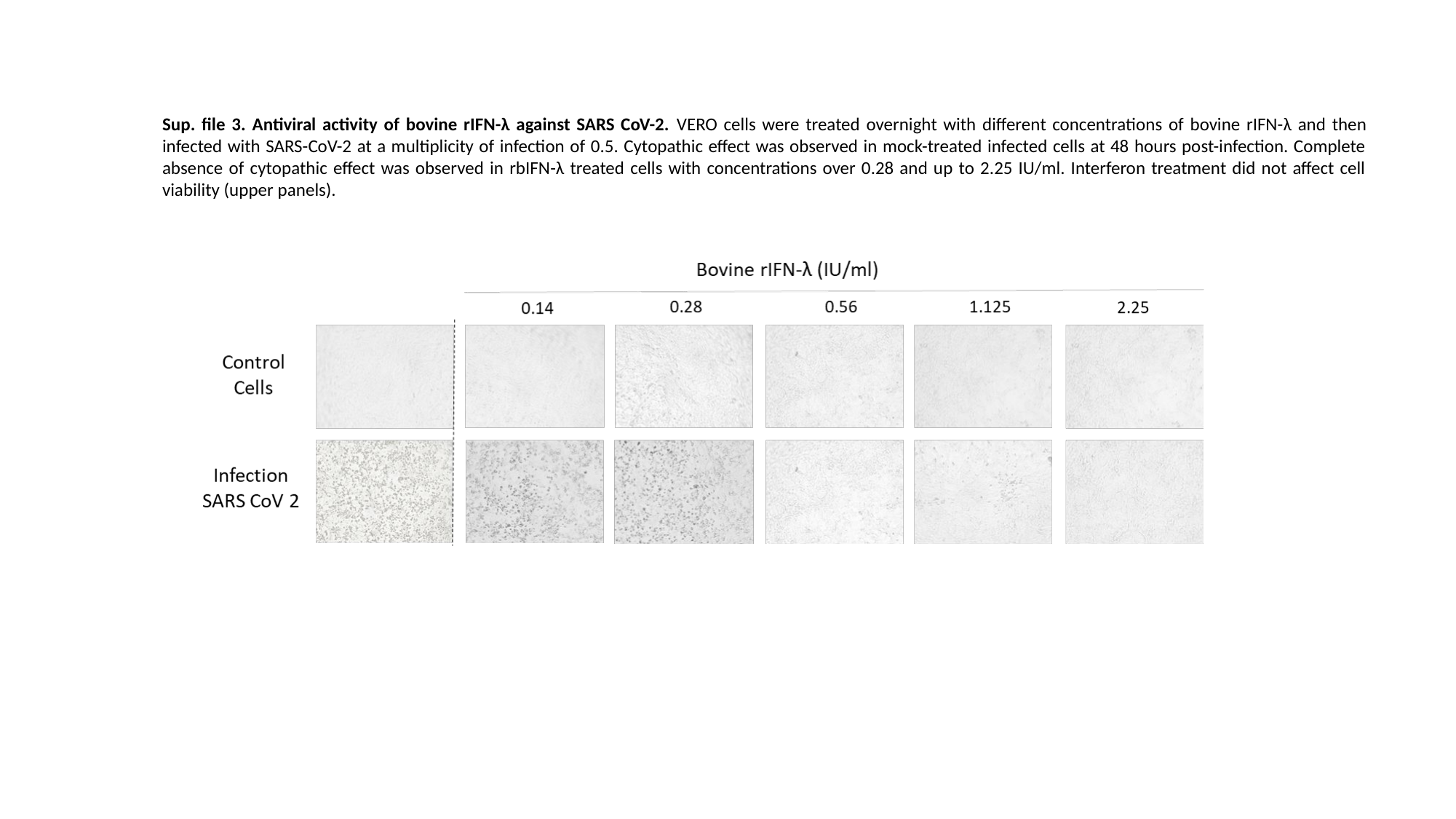

Sup. file 3. Antiviral activity of bovine rIFN-λ against SARS CoV-2. VERO cells were treated overnight with different concentrations of bovine rIFN-λ and then infected with SARS-CoV-2 at a multiplicity of infection of 0.5. Cytopathic effect was observed in mock-treated infected cells at 48 hours post-infection. Complete absence of cytopathic effect was observed in rbIFN-λ treated cells with concentrations over 0.28 and up to 2.25 IU/ml. Interferon treatment did not affect cell viability (upper panels).
